# Creating a Global Community of Practice for Oncofertility

**DOI:** 10.1200/GO.22.00007

**Published:** 2020-03-02

**Authors:** Lauren M. Ataman, Jhenifer K. Rodrigues, Ricardo M. Marinho, João P.J. Caetano, Maurício B. Chehin, Eduardo L. Alves da Motta, Paulo Serafini, Nao Suzuki, Tatsuro Furui, Seido Takae, Yodo Sugishita, Ken-Ichiro Morishige, Teresa Almeida-Santos, Cláudia Melo, Karen Buzaglo, Kate Irwin, W. Hamish Wallace, Richard A. Anderson, Roderick T. Mitchell, Evelyn E. Telfer, Satish K. Adiga, Antoinette Anazodo, Catharyn Stern, Elizabeth Sullivan, Yasmin Jayasinghe, Lisa Orme, Richard Cohn, Rob McLachlan, Rebecca Deans, Franca Agresta, Brigitte Gerstl, William L. Ledger, Rebecca L. Robker, João M. de Meneses e Silva, Lígia H.F. Melo e Silva, Franciele O. Lunardi, Jung R. Lee, Chang S. Suh, Michael De Vos, Ellen Van Moer, Dominic Stoop, Veerle Vloeberghs, Johan Smitz, Herman Tournaye, Ludwig Wildt, Katharina Winkler-Crepaz, Claus Y. Andersen, Brigid M. Smith, Kristin Smith, Teresa K. Woodruff

**Affiliations:** 1 Feinberg School of Medicine, Northwestern University, Chicago, IL; 2 Brazilian Oncofertility Consortium, Belo Horizonte, Brazil; 3 Pró-Criar Medicina Reprodutiva, Belo Horizonte, Brazil; 4 Faculdade de Ciências Médicas de Minas Gerais, Belo Horizonte, Brazil; 5 Huntington Reproductive Medicine and Federal University of São Paulo, São Paulo, Brazil; 6 St Marianna University School of Medicine, Knagawa, Japan; 7 Gifu University Graduate School of Medicine, Gifu, Japan; 8 University Hospital of Coimbra, Coimbra, Portugal; 9 University of Coimbra, Coimbra, Portugal; 10 Clinique Ovo, Montreal, Quebec, Canada; 11 Cancer Knowledge Network, Milton, Ontario, Canada; 12 Edinburgh Royal Hospital for Sick Children, Edinburgh, United Kingdom; 13 Queen’s Medical Research Institute, University of Edinburgh, Edinburgh, United Kingdom; 14 Centres for Fertility Preservation and Integrative Physiology, University of Edinburgh, Edinburgh, United Kingdom; 15 Kasturba Medical College, Manipal University, Manipal, India; 16 Sydney Children’s and Prince of Wales Hospital, Future Fertility, Randwick, New South Wales, Australia; 17 Royal Women’s Hospital, University of Melbourne, Melbourne, Victoria, Australia; 18 University of Technology, Sydney, New South Wales, Australia; 19 Children’s Cancer Centre, Royal Children’s Hospital, Parkville, Victoria, Australia; 20 Kids Cancer Centre, Sydney Children’s Hospital Sydney, New South Wales, Australia; 21 School of Women’s and Children’s Health, University of New South Wales, Royal Hospital for Women, Sydney, New South Wales, Australia; 22 Monash Institute of Medical Research, Prince Henry’s Institute, Clayton, Victoria, Australia; 23 Robinson Research Institute, University of Adelaide, Adelaide, South Australia, Australia; 24 Ceara Blood Center, Centro de Hematologia e Hemoterapia do Ceará, Fortaleza, Brazil; 25 Assis Chateaubri and Maternity School, Federal University of Ceara, Fortaleza, Brazil; 26 Federal University of Ceara and Femini Imagem and Ultravida Clinic, Fortaleza, Brazil; 27 State University of Ceara, Fortaleza, Brazil; 28 Seoul National University Bundang Hospital, Seongnam; 29 Seoul National University College of Medicine, Seoul, Korea; 30 Centre for Reproductive Medicine, Universitair Ziekenhuis (UZ) Brussel, Belgium; 31 Laboratory of Clinical Chemistry and Radioimmunology, UZ Brussel, Brussels, Belgium; 32 Innsbruck Medical University, Innsbruck, Austria; 33 Juliane Marie Centre for Women, Children and Reproduction, University Hospital of Copenhagen, Copenhagen, Denmark

## Abstract

Fertility preservation in the cancer setting, known as oncofertility, is a field that requires cross-disciplinary interaction between physicians, basic scientists, clinical researchers, ethicists, lawyers, educators, and religious leaders. Funded by the National Institutes of Health, the Oncofertility Consortium (OC) was formed to be a scientifically grounded, transparent, and altruistic resource, both intellectual and monetary, for building this new field of practice capable of addressing the unique needs of young patients with cancer. The OC has expanded its attention to include other nonmalignant conditions that can threaten fertility, and the work of the OC now extends around the globe, involving partners who together have created a community of shared effort, resources, and practices. The OC creates materials that are translated, disseminated, and amended by all participants in the field, and local programs of excellence have developed worldwide to accelerate the pace and improve the quality of oncofertility research and practice. Here we review the global oncofertility programs and the capacity building activities that strengthen these research and clinical programs, ultimately improving patient care.

## INTRODUCTION

Survival rates among young patients with cancer have steadily increased over the past three decades, in part because of the development of more effective cancer treatments.^1,2^ Today, both women and men can look forward to life after cancer; however, many may face the possibility of infertility as a result of the disease itself or these lifesaving treatments. Established in 2007 as part of a National Institutes of Health center grant, the Oncofertility Consortium (OC) is an interinstitutional, interdisciplinary consortium to expand research in fertility loss in patients with cancer, accelerate clinical translation of fertility preservation techniques, and address the complex health care and quality-of-life issues that concern young patients with cancer whose fertility may be threatened by their disease or its treatment.^3–7^ The term oncofertility was originally coined to describe a new discipline that bridges oncology and reproductive medicine to discover and apply new fertility preservation options for young patients with cancer. However, as the OC worked to create fertility preservation technologies and clinical oncology management plans for patients with cancer, it became clear that fertility concerns resulting from nonmalignant diseases and iatrogenic causes were much broader than just those associated with cancer. GI diseases, rheumatologic disorders, nonmalignant hematologic conditions (most prominently β thalassemia), neurologic disorders, renal disorders, gynecologic conditions, and metabolic diseases can all adversely affect fertility. By expanding its scope, the OC now ensures that all patients facing a disease or treatment that limits reproductive function can benefit from the findings of basic and clinical reproductive research. The word oncofertility was created when few options were available and now provides terminology for a medical field at the intersection of many iatrogenic causes of infertility.

To facilitate sharing of knowledge and resources, the OC formed the National Physicians Cooperative, which today represents > 60 centers across the United States that provide oncofertility services to men and women, as well as 19 centers focused on pediatric patients.^8^ Since its inception, the OC has aimed to involve more partners to create a nationwide community of shared resources and practices, with the ultimate goal of improving patient care. Today, there is wide acceptance that partnerships that bring research and clinical teams together catalyze progress, and the global partnerships discussed here are moving quickly to provide broad reproductive care to anyone experiencing an iatrogenic impact on reproduction, fertility, or sexuality.^2^

**FIG 1:**
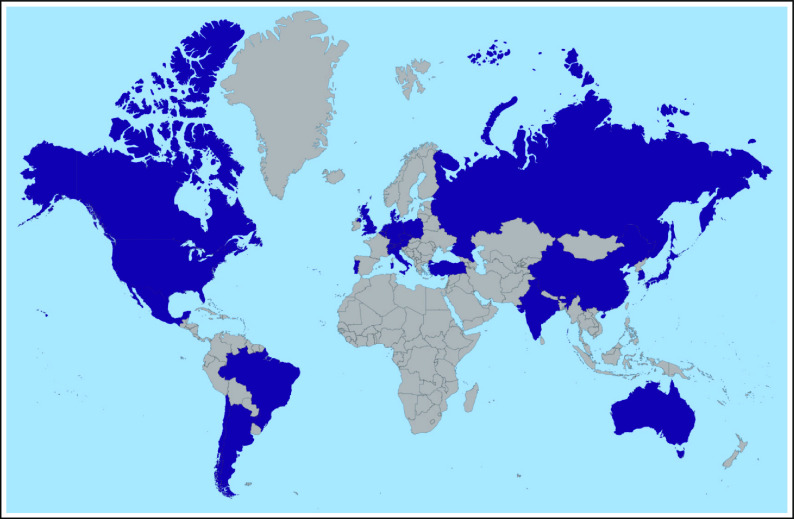
Map of countries (purple) represented in Oncofertility Consortium global partners network.

There are currently 19 countries engaged in the global oncofertility community (Fig 1), and the hope is to continue to grow and expand these relationships. As individual centers of excellence realize the intrinsic value in joining a larger global community of shared practices, the global oncofertility community is strengthened, and the OC and its partners can begin to engage international advocacy groups, governments, and others. Advances in technology, such as video conferencing, connect researchers, scientists, and patients from around the globe, making real-time sharing of scientific results and medical best practices possible and raising awareness among diverse stakeholders. By breaking free of the outdated, reclusive nature of the scientific community, and being willing to share successes, failures, challenges, and triumphs, the OC is building the field of oncofertility, from bench to bedside to babies. Engaging all stakeholders, beyond scientists and clinicians, will continue to broaden the influence of the community and improve care for all patients.

**FIG 2:**
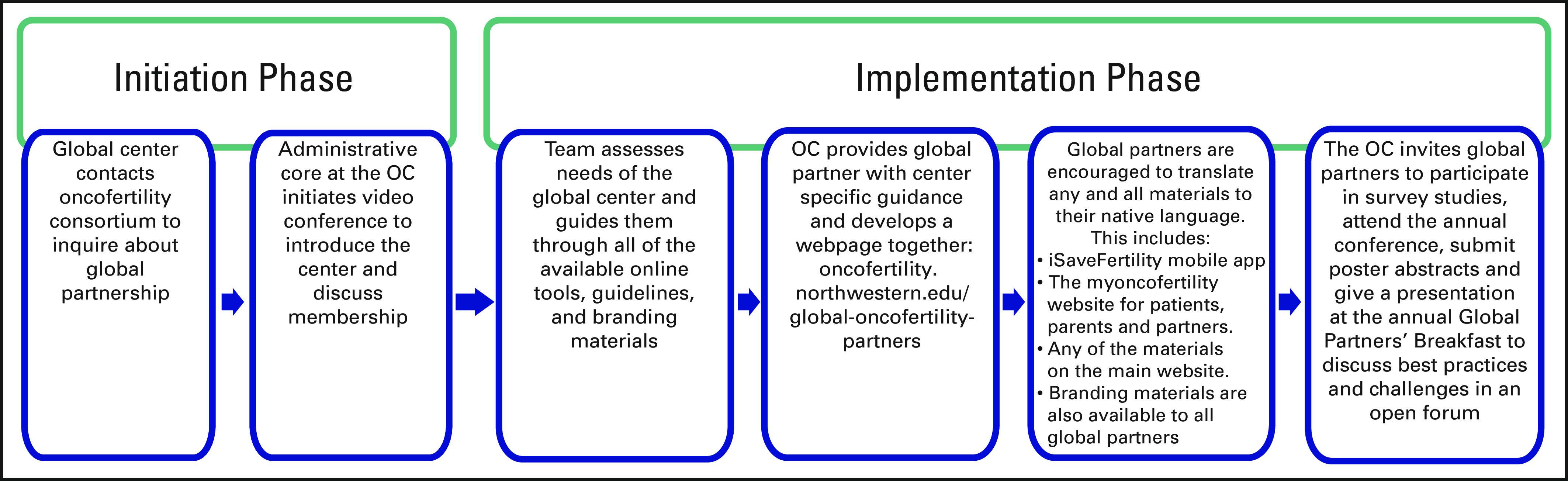
Flow chart: becoming global partner. OC, Oncofertility Consortium.

Global partners of the OC receive tools and guidance to set up their own local consortia (Fig 2). The administrative core of the OC at Northwestern University serves global partners in its efforts to build and expand its existing services and outreach. All of the OC materials on the main website (oncofertility.northwestern.edu) and other online resources are made available to global partners, including the iSaveFertility mobile app (savemyfertility.org), patient navigator tools (preservefertility.northwestern.edu), decision aids (myoncofertility.org), and Repropedia (http://www.repropedia.org), an online reproductive lexicon. Branding materials are available at oncofertility.northwestern.edu/branding-materials. Global partners are encouraged to translate these materials and disseminate them to international audiences, while in turn providing our team with new content and links to include on the main OC Web site (oncofertility.northwestern.edu). A complete list of active global partners and the work they have contributed can be found here: http://oncofertility.northwestern.edu/global-oncofertility-partners. Making these materials available to all partners fosters interaction and a shared language and purpose among diverse groups, which enables our global partners to apply these resources, methodologies, and other experiences in the field. Guidelines are not within the scope of the consortium, but the OC does provide a site for aggregation and dissemination of guidelines from formal medical societies, and guidelines for our field from oncology, fertility, pediatric, and nursing specialty groups from around the globe can be found in one central location (http://oncofertility.northwestern.edu/ODT-web-portal). Establishing a strong global network not only drives the collaborative nature of the OC, but also helps global partners build their own consortia and fertility preservation networks.^9^

**FIG 3:**
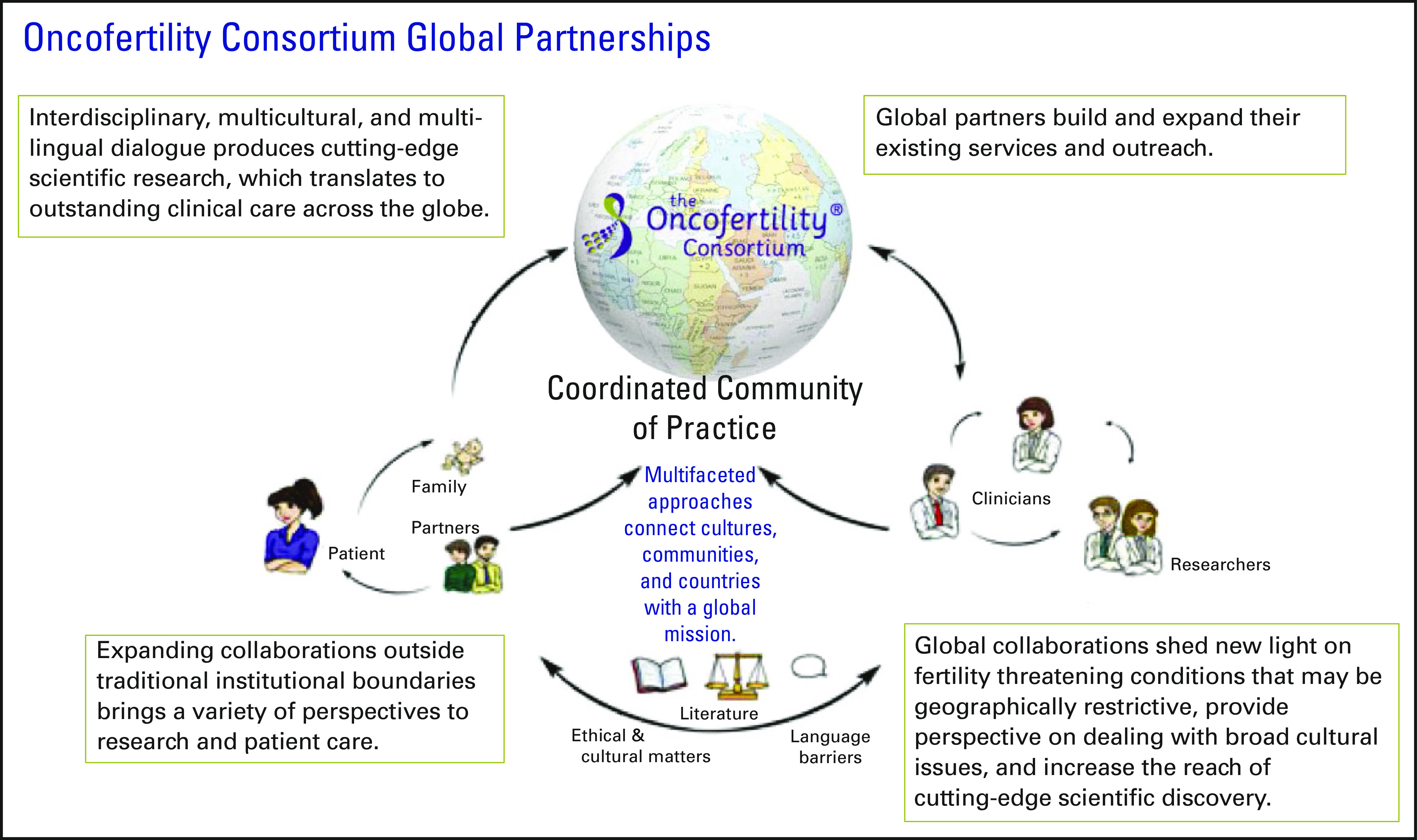
Oncofertility Consortium global partnerships model.

As part of the global partners model (Fig 3), the OC works with multidisciplinary reproductive specialists from all over the world in an effort to better serve children, adolescents, and young adults with cancer and other fertility-threatening diseases. Global collaborations shed new light on fertility-threatening conditions in other countries, reveal new perspectives on addressing broad cultural issues, and increase the reach of cutting-edge scientific discoveries. It is this interdisciplinary, multicultural, and multilingual dialogue that the OC thrives on to continue to advance scientific research and translate discovery to outstanding clinical care around the globe.

## CASE FOR GLOBAL NETWORKING TO ESTABLISH REPRODUCTIVE HEALTH STANDARDS OF CARE

In recent decades, globalization has extended the reach of research universities,^10^ with striking success in advancing knowledge.^11^ Similarly, extending the oncofertility network beyond the confines of the United States and into the international realm not only advances knowledge and promotes discovery, but also provides uniform access for all experts and their unique perspectives. These collaborations benefit the scientific community by accelerating the pace of discovery and decreasing the time to clinical application. The capacity for making landmark breakthroughs in the field is enhanced. Furthermore, research has shown that the trend toward international collaboration and network building attracts attention to issues and leads to a greater number of publications and greater support for basic research and clinical studies.^12^ Global partnerships, including research collaborations and joint authorships, strengthen the dissemination of scientific results; as the size of the audience increases, so does the reach and influence of the research, thus increasing the potential translational impact the research may have on future patient care.

Because of the intrinsic value in creating diverse networks and collaborations, the OC continues its efforts to connect local centers of excellence and create a strong global network of diverse collaborators, many of whom may not have worked together otherwise. The OC supports interaction between global and local partners to create momentum for clinical activities (shared protocols and patient case studies, inclusion of allied health professionals), research (sharing results, both failures and successes, in ways that hasten work), and meeting patient needs (educational Web sites, patient decision tools, patient navigator). By facilitating these interactions, the OC ensures the coordinated effort of the global oncofertility community in conducting cutting-edge research that can continue to be rapidly translated to the clinic and establish an evidence-based standard of care.

Today, many global partners are contributing to the worldwide oncofertility network (Table 1). Here, we describe six partners that have been actively engaged with the OC and have made notable contributions to the field, with programs that are uniquely tailored to the needs of patients in their respective countries. The OC provided the basic foundation, insights, and resources for establishing these centers, which serve to extend fertility preservation research efforts; in return, these global partners share their diverse perspectives, experiences, scientific findings, and attitudes with the OC and other global partners to enrich patient care.

**TABLE 1:**
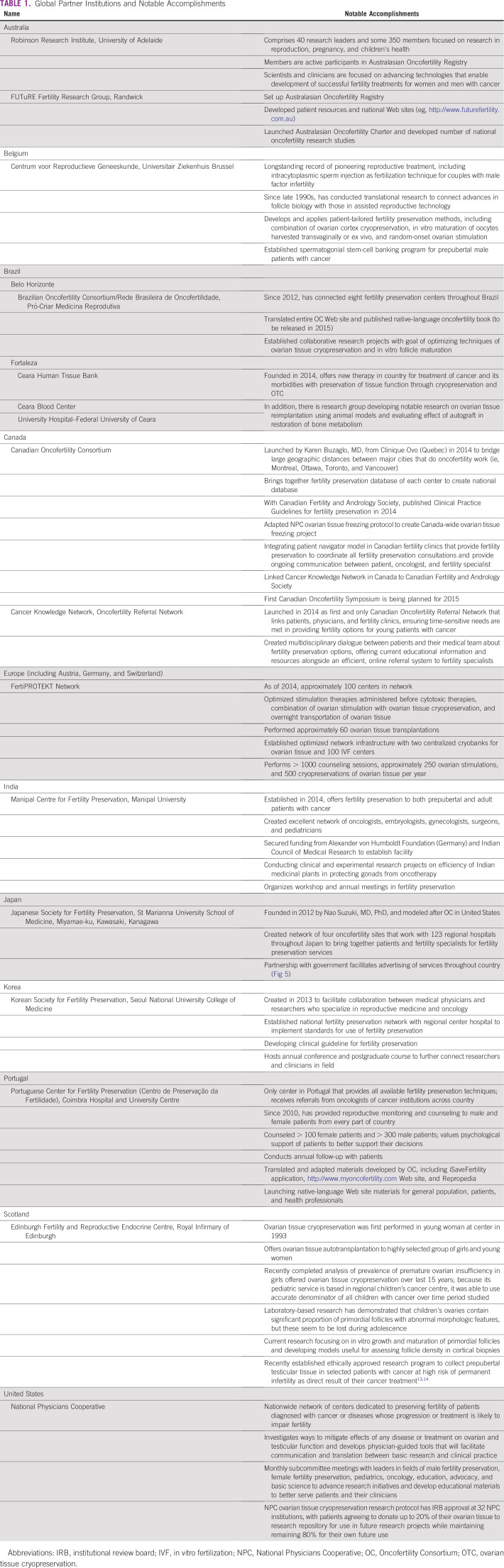
Global Partner Institutions and Notable Accomplishments

### Australasia

In Australia and New Zealand, a number of cancer and fertility groups have developed specific oncofertility services and are undertaking research studies to bridge the intellectual, disciplinary, and logistic gaps between reproductive medicine and oncology. The Fertility Society of Australia established a special interest group in 2008 with the aim of facilitating collaborative research and improving communication and education between cancer and fertility clinicians.

The Australasian OC was established in 2014 and is committed to interdisciplinary innovation. The consortium endeavors to support the collaborative efforts of cancer and fertility clinicians in Australasia (Australia and New Zealand) to improve oncofertility practice and services. The consortium developed the Australasian Oncofertility Charter, which outlines the gold-standard model for care to be implemented at each service. Working closely with consumer groups, > 30 resources on the topics of fertility preservation, sexual health, and sexual dysfunction have been made available on our Web site (http://www.futurefertility.com.au). They are currently in the process of developing e-learning tools that will also be available for clinicians early in the new year.

The FUTuRE (Fertility Understanding Through Registry and Evaluation) Fertility Team is a binational group of researchers who set up the Australasian Oncofertility Registry using a Web-based platform. In 2015, this registry will start collecting oncofertility population data from patients with cancer in Australia and New Zealand ages 0 to 45 years. These data will reveal referral patterns for fertility preservation, uptake, and use of fertility preservation, reproductive risk based on annual follow-up of patients, and pregnancy outcomes (both natural and assisted in patients with cancer). This will be the first population-based national oncofertility registry and will produce invaluable insights into oncofertility care that are highly relevant to the efforts of international colleagues.

### Brazil

**FIG 4:**
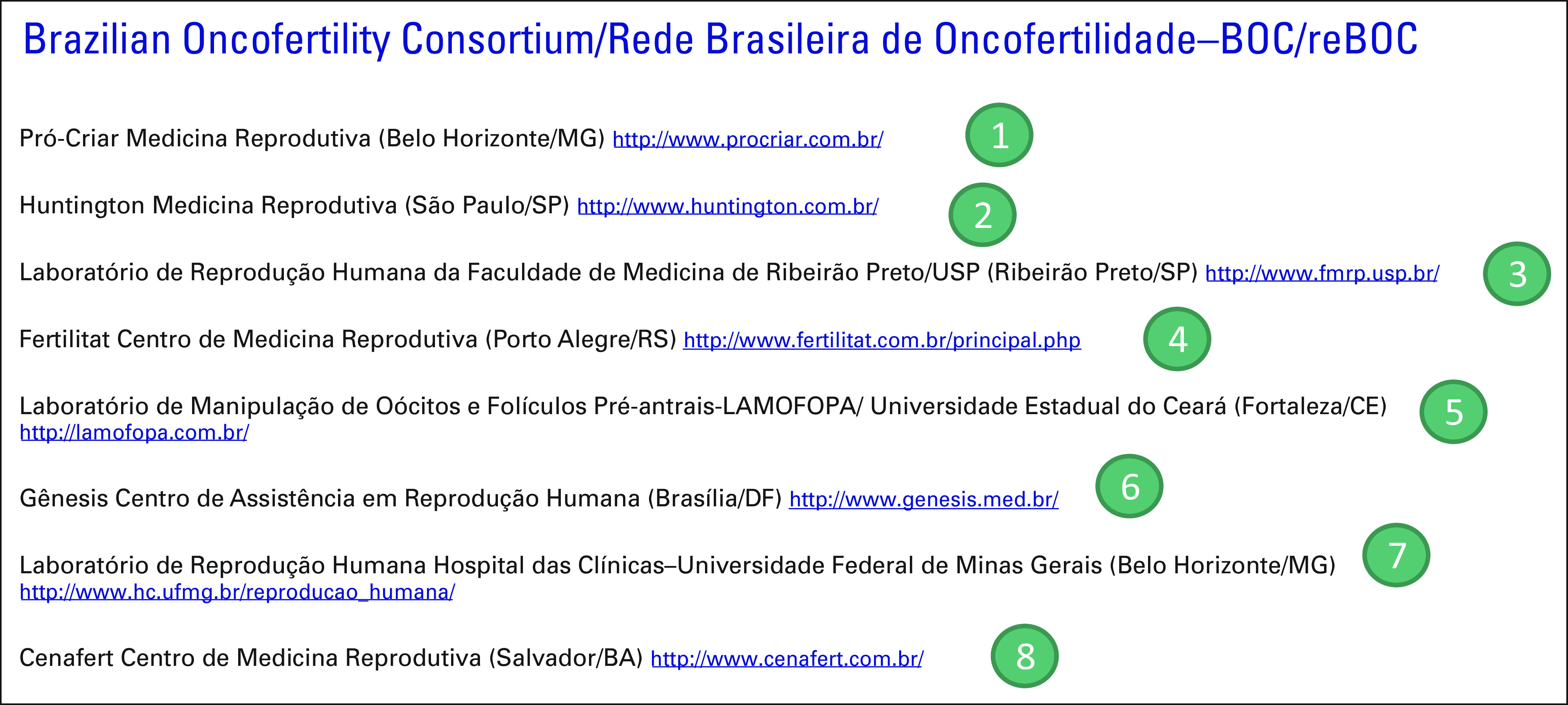
Brazilian Oncofertility Consortium.

The Brazilian OC (BOC) was officially created in 2012. Known nationally in Brazil as Rede Brasileira de Oncofertilidade and coordinated by Jhenifer K. Rodrigues, PhD, the group is a network of professional members who share experiences, materials, research protocols, and ideas and engage in collaborative research projects. The network includes the Pró-Criar Medicina Reprodutiva and Human Reproduction Laboratory/Federal University of Minas Gerais from the state of Minas Gerais (southeast), Huntington Medicina Reprodutiva and Medical School of Ribeirão Preto/University of São Paulo from São Paulo state (southeast), Fertilitat Centro de Medicina Reprodutiva from Rio Grande do Sul (south), Laboratory of Oocytes and Preantral Follicles Manipulation/State University of Ceará from Ceará (north), Gênesis Centro de Assistência em Reprodução Humana (Brasília, Federal District; central eastern), and Cenafert Centro de Medicina Reprodutiva from the state of Bahia (northeast; Fig 4). The BOC has increased the level of awareness and national discussion about the fertility preservation options of patients with cancer. Through the BOC, patients with cancer are directed to key centers of assisted reproduction, which offer the most advanced research protocols for cryopreservation of semen, oocytes, embryos, and ovarian tissue to preserve fertility. Patients also receive psychological treatment to aid in the decision-making process.

In Fortaleza, the group was founded in 2014. It offers new therapy in the region for the treatment of cancer and its morbidities, with preservation of tissue function through ovarian tissue cryopreservation. In addition, there is a group developing notable research on ovarian tissue reimplantation using animal models and evaluating the effect of autograft in restoration of bone metabolism.

In just 2 years, the BOC has formed connections with other global oncofertility groups, including the Korean Society for Fertility Preservation (KSFP) and the Japan Society for Fertility Preservation (JSFP). The BOC has also collaborated on publications and research projects with the OC in the United States. The group has already published a native-language oncofertility book with contributions from experts across Brazil and some from abroad. Now the group is working on developing more native-language resources to further connect with patients in Brazil. Efforts in Brazil are being made to bridge the gap between oncologists and reproductive specialists to help inform patients with cancer about their fertility preservation options and reproductive future.

### Europe

Working from an initiative in the Departments for Gynecological Endocrinology and Reproductive Medicine at the Universities of Heidelberg and Bonn in Germany, the FertiPROTEKT network was founded in May 2006. Two years later, the network was extended to Austria and Switzerland to include not only universities but also private centers offering fertility preservation.

Since January 2014, approximately 100 centers across Germany, Austria, and Switzerland have joined the FertiPROTEKT network. Similar to the OC, the FertiPROTEKT network seeks to improve the standard of care for all patients by implementing standardized protocols and methods of quality control. The main achievements of the network are the optimization of stimulation therapies administered before cytotoxic therapies, the combination of ovarian stimulation with ovarian tissue cryopreservation, and the establishment of overnight transportation of ovarian tissue. Thus far, network members have performed and analyzed approximately 60 ovarian tissue transplantations. Furthermore, an optimized network infrastructure has been established, with two centralized cryobanks for ovarian tissue and 100 in vitro fertilization centers, which perform > 1,000 documented counseling sessions, 250 ovarian stimulations, and 500 cryopreservations of ovarian tissue per year. The homepage of the FertiPROTEKT network (http://www.fertiprotekt.com), which has > 100,000 visitors per year, can be accessed in German or English. Annual meetings involving all participating centers guarantee consistent therapy standards among the centers. The therapy standards of the network are constantly updated and published.^15^

### Japan

In November 2012, the JSFP was founded as a nonprofit corporation (http://www.j-sfp.org) with the aim of improving both the survival and quality of life of young patients with cancer in Japan. As part of this effort, the society is currently building a network for coordinating health care providers, using the OC as a guide, to provide accurate information about fertility preservation to young patients with cancer in a timely manner. The JSFP has hosted a number of conferences aimed at attracting the attention of all health care providers who are concerned with fertility preservation and oncofertility. To date, these conferences have been attended by approximately 280 clinicians, nurses, pharmacists, scientists, and others.

**FIG 5:**
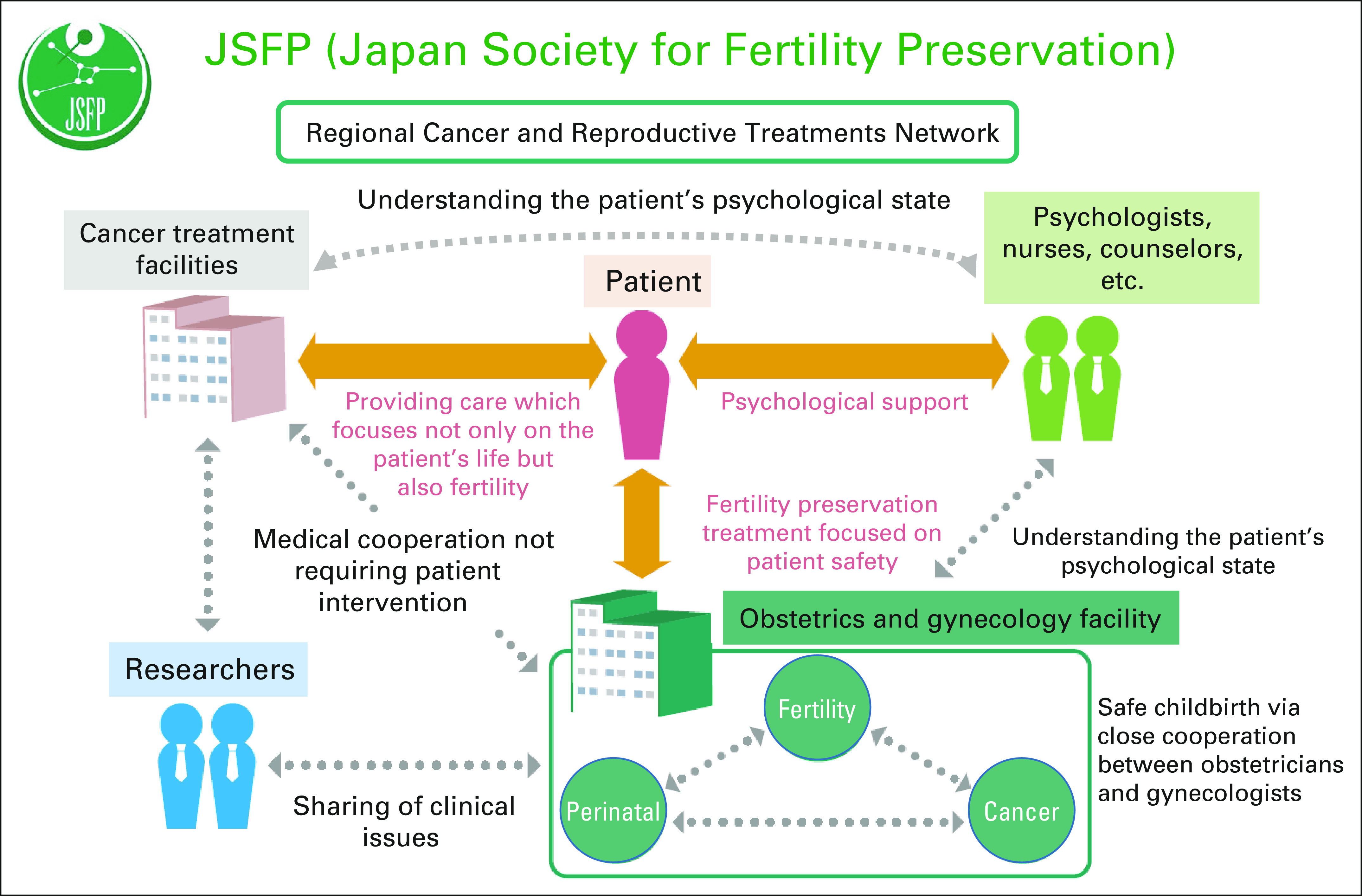
Japan Society for Fertility Preservation.

As shown in Figure 5, the JSFP is aiming to build a health care coordination system capable of quickly providing information on fertility preservation to patients with cancer and their families within a specific local community. In 2013, an initial self-contained regional health care coordination system was established by Ken-Ichiro Morishige and Tatsuro Furui, MD, PhD, at the Gifu University School of Medicine, called Gifu Patients, Oncologists, and Fertility Specialists, in collaboration with the Gifu Prefectural Government (Fig 5). Since then, similar regional health care networks have been established in Okayama, Nagasaki, Fukuoka, and Okinawa Prefectures, with the intention to achieve nationwide operation.

In 2014, the JSFP used a Health and Labor Sciences research grant to develop the Clinical Practice Guidelines for Preservation of Fertility in Breast Cancer Patients in Japan (research representative, Chikako Shimizu, MD, Breast and Medical Oncology Division, National Cancer Center Hospital). The JSFP has also secured funding from a Health and Labor Sciences research grant for a study titled “Development of a Psychological Support System for Fertility Preservation Aimed at Achieving Improvements for Young Cancer Survivors” (research representative, Nao Suzuki, MD, PhD, St Marianna University School of Medicine). The JSFP is also working to initiate a clinical study titled “Oncofertility! Psychological Education and Couple Enrichment (O!PEACE) Therapy: An Intervention Study Protocol for a Randomized Controlled Trial in Japan.” The aim of this study is to examine whether psychotherapy can reduce concerns about fertility, alleviate psychological distress, and improve communication between patients with cancer and their partners. To expand its efforts, the JSFP is actively engaged in various activities with OC Japan, in cooperation with the OC, which are aimed at expanding oncofertility programs in Asia in association with the KSFP and the Fertility Preservation Society of India (FPI).

### Portugal

The Portuguese Centre for Fertility Preservation (at Coimbra Hospital and University Centre) was created in 2010 to meet the reproductive needs of patients undergoing treatments that are possibly fertility threatening. Although male fertility preservation had been available in several public institutions since the 1990s, female fertility preservation options were not available in Portuguese public practice. This center is the only one in the country that provides all fertility preservation techniques to both men and women.

**FIG 6:**
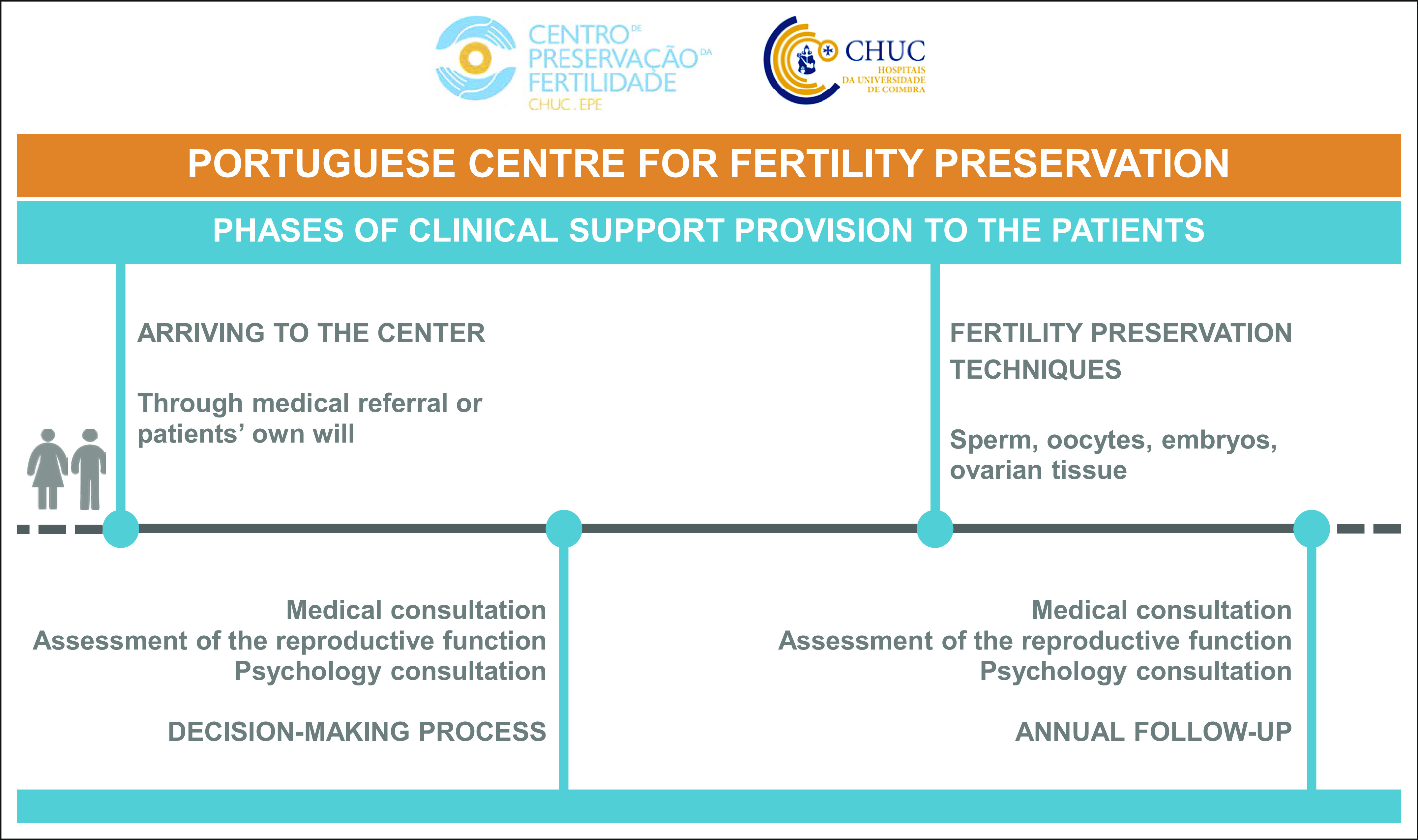
Portuguese Centre for Fertility Preservation.

The team at the center includes six physicians, one embryologist, one psychologist, and one pharmacist. Through a multidisciplinary approach, the main goal is to provide reproductive monitoring and counseling to male and female patients undergoing gonadotoxic treatments. This team supports the patients' decision-making process about fertility preservation and supports their reproductive decisions after treatment through regular follow-up consultations (Fig 6).

Although most of the patients with cancer are referred to the center by their oncologists, a significant number of them ask for a consultation on their own. Thus, another goal of this center is to try to better inform patients, health professionals, and the general population about the impact of cancer on fertility, the techniques available for fertility preservation, and how to assemble a team that can provide patients counseling and assistance in making decisions. To achieve this goal, the center has developed information fact sheets for patients and, in collaboration with the Portuguese Society for Reproductive Medicine, has organized postgraduate courses. Some materials from the OC (eg, iSaveFertility app, Web site http://www.myoncofertility.org, and Repropedia tool) were translated into Portuguese to allow the center to better inform the general population and health professionals about fertility preservation, thereby aiding in the decision-making process. The Portuguese Centre for Fertility Preservation Web site is about to be launched and will include information and tools tailored specifically to Portuguese patients and health professionals. The center is working with the Portuguese Society of Reproductive Medicine to develop a network with other public Portuguese institutions that can provide fertility preservation techniques to improve patient referral processes. Finally, research projects are also being developed to examine the fertility preservation decision-making process, the impact of this decision on future individual adaptation, the effect of cancer treatments on patients' reproductive function, and new fertility preservation techniques. Specifically in 2015, the main goals of the Portuguese Centre for Fertility Preservation are: to develop the first telemedicine network in oncofertility so that patients and oncologists anywhere can reach out the center team to enroll in multidisciplinary consultations to make more informed, shared, and quick decisions about fertility preservation, to produce and disseminate oncofertility decision aids specifically to the pediatric population and to pediatricians, and to create and implement protocols for the cryopreservation of ovarian and testicular tissues for prepubertal patients.

### Korea

The KSFP was established in 2013 (http://www.ksfp2013.org) to facilitate collaboration between medical physicians and researchers who specialize in reproductive medicine and oncology. The ultimate purpose of the society is to help patients who are undergoing treatments that will affect their fertility and reproductive function. The goals of the KSFP are academic education, networking, advocacy, discussion, and development of standard protocols for fertility preservation.

**FIG 7:**
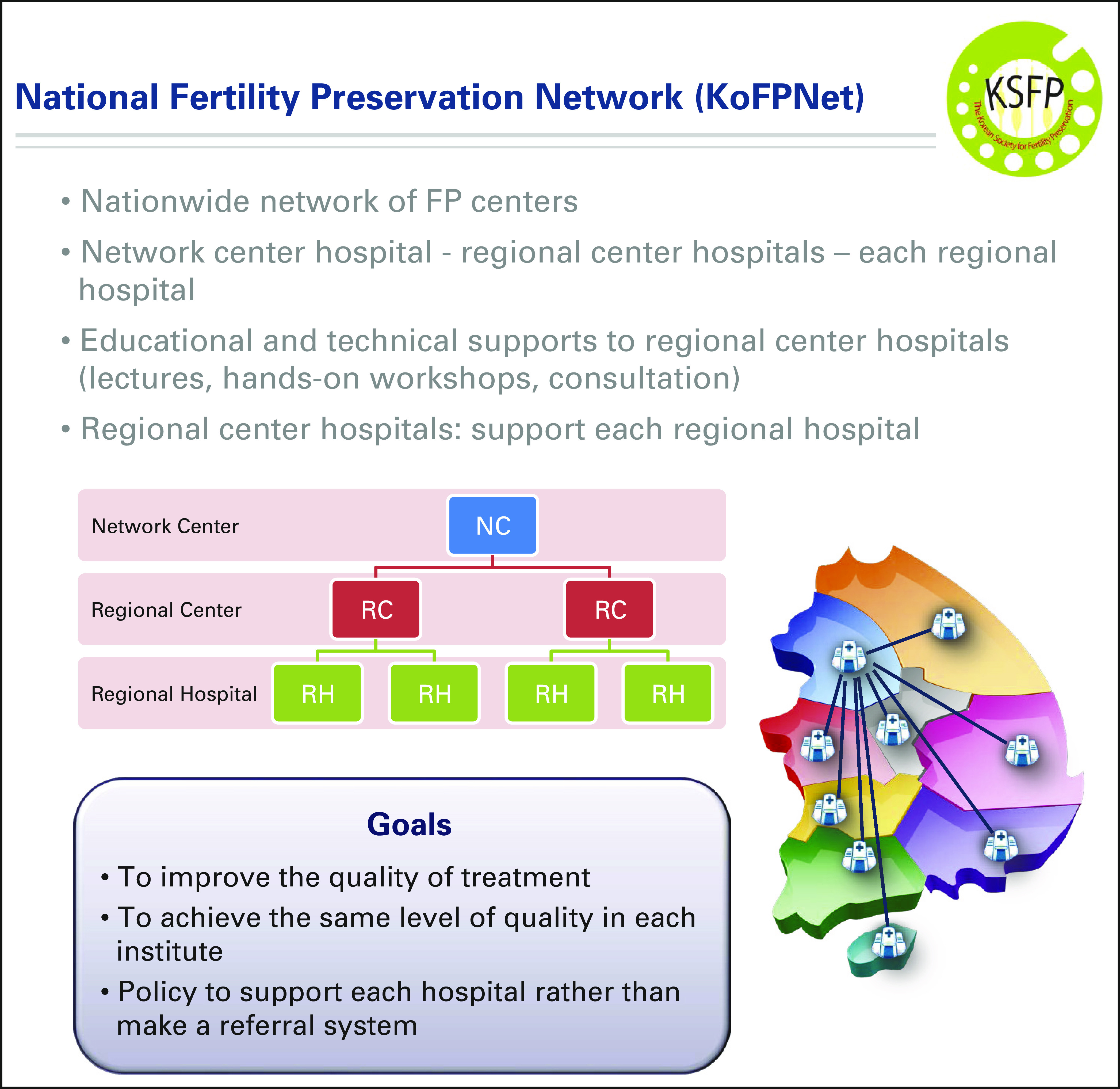
Korea National Fertility Preservation Network.

The KSFP holds an annual conference and postgraduate course and has established a national fertility preservation network. The Korean National Fertility Preservation Network is a nationwide network of fertility preservation centers. It has a three-tier structure: national center hospitals, regional center hospitals in each region, and regional hospitals (Fig 7). The KSFP provides education and technical support to the regional center hospitals of the network, including lectures, hands-on workshops, and consultation. In turn, the regional center hospitals support their associated regional hospitals. The goals of this network are to improve the quality of treatment and to achieve the same level of quality in each institution.

As a global partner of the OC, the KSFP collaborates with all other global partners, sharing information and experience. With regard to improving oncofertility research and clinical programs in Asia, the KSFP collaborates with the JSFP in Japan and supports the plan to establish an Asian oncofertility society.

## BARRIERS, CHALLENGES, AND OPPORTUNITIES FOR COLLABORATIVE FUTURE IN ONCOFERTILITY

The nature of oncofertility—bridging reproductive health and oncology, basic science and clinical research, medical and social science, and the academy and the public—means that its work relies on collaboration. The collective knowledge and experiences of all OC partners and stakeholders, both national and international, are what drive the success of the global oncofertility effort. There are certainly challenges and barriers to success that the OC has faced, which in part are the ordinary issues of time and distance. However, the common goals for the groups have instilled a sense of unity among team members. We learn from one another and in so doing catalyze individual work. Country-to-country restrictions (eg, on embryo banking or use of gestational carrier) may limit activity in one region, but a common understanding regarding what is possible in another area of the work may provide insight and opportunities to overcome these local barriers. Ultimately, there is no precise formula to success. Because of a variety of factors, what works for one center may not work for another. Our success comes from simply engaging an inspired individual or group of individuals to act and our collective interest in the people with iatrogenic fertility concerns that we serve. The OC has made it possible for individual practices to tap into a worldwide network of knowledge, experience, and discovery to improve patient care. OC partners communicate and work across disciplinary, institutional, and geographic boundaries. Training the next generation of oncofertility collaborators to undertake this multidisciplinary, multinational approach, as well as to engage all stakeholders, is essential to ensure continued rapid transfer of sound science to quality medical practice. With each new partner, particularly those outside the traditional institutional boundaries, a variety of new scientific, cultural, ethical, and personal perspectives are brought to bear on oncofertility research and patient care. Multifaceted approaches to connect cultures, communities, and countries show the most promise for future success.^16–18^ By engaging global partners in an inclusive approach to fertility preservation research and clinical care, the OC will continue to create cohesive and highly effective communities of oncofertility practice.

## Appendix

Brazilian Oncofertility Consortium representative center members: Ana Carolina Japur de Sá Rosa e Silva (Faculdade de Medicina de Ribeirão Preto [FMRP]/University of São Paulo [USP]), Jacira Campos (FMRP/USP), Álvaro Petracco (Fertilitat), Mariângela Badalotti (Fertilitat), Ricardo Azambuja (Fertilitat), José Ricardo Figueiredo (O Laboratório de Manipulação de Oócitos e Folículos Ovarianos Pré-antrais [LAMOFOPA]), Ana Paula Ribeiro Rodrigues (LAMOFOPA), Bruno Ramalho (Gênesis), Adelino Amaral (Gênesis), Joaquim Lopes (Cenafert), and Fernando Reis (Universidade Federal de Minas Gerais). Canadian representatives: Cancer Knowledge Network produced by Multimed. Japanese Society for Fertility Preservation representatives: Kouhei Sugimoto, Tomoe Koizumi, Nobuhito Yoshioka, and Chie Nishijima. Korean Society for Fertility Preservation: Dong Young Noh, Byung Seok Lee, Kyung Joo Hwang, Byung Chul Jee, Ki Chul Kim, Tak Kim, Taek Hoo Lee, DooSeok Choi, Woo Sik Lee, Sung Won Kim, Hee Dong Chae, Tae You Kim, Dong Ryul Lee, Chung Hyon Kim, and Seul Ki Kim. Members of Centro de Preservação da Fertilidade do Centro Hospitalar e Universitario de Coimbra: Merck SA (Portugal), Professor Carlos Freire de Oliveira. Partners at FertiPROTEKT: Michael von Wolff and Ludwig Wildt. Members of the National Physicians Cooperative are listed on the Oncofertility Web site (http://oncofertility.northwestern.edu/) and are gratefully acknowledged for their work on this topic.
